# Psychological flexibility as a predictor of fear of happiness in young adults: the role of life satisfaction

**DOI:** 10.3389/fpsyg.2026.1807612

**Published:** 2026-06-03

**Authors:** Kadriye Karagülmez Gül, Nergüz Bulut Serin

**Affiliations:** Education Faculty, Department of Guidance and Psychological Counseling, Institute of Graduate Studies & Research, European University of Lefke, Lefke/North Cyprus, Mersin, Türkiye

**Keywords:** experiential avoidance, fear of happiness, life satisfaction, positive affect regulation, psychological flexibility

## Abstract

Psychological flexibility is considered a central process within the framework of Acceptance and Commitment Therapy (ACT); however, its relationship with fear of happiness remains insufficiently understood. The present study aims to examine the associations between psychological flexibility, fear of happiness, and life satisfaction in young adults, and to investigate whether life satisfaction functions as a potential mediating mechanism in this relationship. In this context, the study seeks to evaluate whether fear of happiness can be explained through process-based regulatory mechanisms. A cross-sectional design was employed with a sample of 370 university students aged 19–26. Data were collected using the Acceptance and Action Questionnaire-II, the Fear of Happiness Scale, and the Satisfaction with Life Scale. A moderated mediation analysis was conducted using PROCESS Model 59 with 5,000 bootstrap samples. The results indicated that psychological flexibility was negatively associated with fear of happiness and positively associated with life satisfaction. Although life satisfaction was significantly related to both variables, the small magnitude of the indirect effect and the absence of a consistent indirect effect at the model level suggest that life satisfaction does not function as a strong mediating mechanism in this relationship. In addition, the index of moderated mediation was not statistically significant, indicating that the indirect effect did not differ reliably across groups. These findings suggest that psychological flexibility is directly associated with fear of happiness and that this relationship is relatively independent of global evaluations of life. From an ACT perspective, maladaptive responses to positive emotions appear to be more closely related to low psychological flexibility and associated experiential avoidance processes than to general cognitive evaluations of life circumstances. Overall, the results highlight the central role of process-based mechanisms in understanding fear of happiness and underscore the importance of enhancing psychological flexibility in interventions aimed at reducing maladaptive beliefs about positive emotional experiences.

## 1 Introduction

Young adulthood is recognized as a critical developmental period characterized by identity exploration, increasing autonomy, and the pursuit of meaningful life goals (Arnett, 2000). During this phase, individuals encounter a range of academic, emotional, and interpersonal challenges that may significantly influence psychological wellbeing and life satisfaction. Understanding the psychological processes that shape how young adults respond to both negative and positive emotional experiences is therefore of particular importance.

Although previous research has documented significant associations between psychological flexibility, fear of happiness, and life satisfaction, the mechanisms through which reduced psychological flexibility is associated with fear of happiness remain insufficiently understood. In particular, to our knowledge, no study has empirically examined whether life satisfaction functions as a mediating mechanism linking psychological flexibility and fear of happiness within an Acceptance and Commitment Therapy (ACT) framework.

Recent research has examined the mediating role of psychological inflexibility in the relationship between aversion to happiness and life satisfaction (Teke and Tunca, 2025). However, these models primarily conceptualize psychological inflexibility as an outcome of fear of happiness rather than as a primary predictor, and have not directly examined whether life satisfaction functions as a mediating mechanism linking psychological flexibility to fear of happiness within an ACT framework. Addressing this gap is important for clarifying whether maladaptive responses to positive emotional experiences are primarily driven by reduced psychological flexibility (i.e., experiential processes) or by broader cognitive evaluations of one's life.

Within the framework of ACT, psychological inflexibility is conceptualized as a core transdiagnostic process characterized by experiential avoidance, cognitive fusion, and rigid behavioral patterns that restrict adaptive engagement with internal experiences and value-consistent actions (Hayes et al., 2006; Kashdan and Rottenberg, 2010). Empirical studies have shown that higher psychological flexibility is associated with lower psychological distress, greater resilience, and more adaptive emotional functioning (Fletcher and Sarkar, 2013; Southwick et al., 2014; Tugade and Fredrickson, 2004). These findings highlight the central role of experiential processes in shaping psychological outcomes and suggest that maladaptive responses to positive emotional experiences, such as fear of happiness, may be rooted in reduced psychological flexibility, reflected in deficits in emotion regulation and experiential openness.

Recent experimental evidence further supports the role of psychological flexibility as a modifiable regulatory process underlying emotional functioning. For example, ACT-based self-help interventions have been shown to significantly increase psychological flexibility while reducing depression, anxiety, and stress among university students (Köksal and Topkaya, 2025). These findings indicate that psychological flexibility represents not only a correlational construct but also a dynamic and intervention-sensitive mechanism that can directly influence how individuals respond to both negative and positive emotional experiences.

From this perspective, fear of happiness can be understood as a form of experiential avoidance directed toward positive affect, whereby individuals perceive positive emotional states as potentially threatening or aversive and attempt to suppress or avoid them (Gilbert et al., 2014; Joshanloo, 2013). Fear of happiness refers to experiencing negative affect, such as fear, anxiety, guilt, or discomfort, when happiness is anticipated, experienced, or expressed (Joshanloo, 2024). Consistent with this view, fear of happiness can be conceptualized as an aversion to hedonic positive affect rather than to broader or more abstract forms of well-being. Cultural beliefs may further reinforce these tendencies, particularly in contexts where expressions of happiness are viewed as socially undesirable or likely to be followed by negative consequences (Joshanloo et al., 2014).

Life satisfaction, in contrast, represents a global and cognitively mediated evaluation of one's overall quality of life (Diener et al., 1985). Prior research indicates that individuals with higher psychological flexibility tend to report greater life satisfaction, whereas individuals who exhibit fear of happiness often report lower levels of life satisfaction due to their avoidance of positive emotional experiences (Joshanloo and Weijers, 2014; Kashdan and Rottenberg, 2010). Together, these findings suggest that life satisfaction may represent a cognitive domain through which experiential processes influence individuals' responses to positive affect.

From an ACT perspective, reduced psychological flexibility is expected to increase experiential avoidance processes, which may influence both individuals' evaluations of their lives and their responses to positive emotional experiences. Specifically, experiential avoidance may reduce life satisfaction by limiting engagement with meaningful and value-consistent activities while simultaneously contributing to the development of fear of happiness by fostering negative beliefs about positive affect. Accordingly, life satisfaction is conceptualized in this study as a potential cognitive pathway through which levels of psychological flexibility may be associated with fear of happiness. This conceptualization distinguishes life satisfaction from outcome variables and positions it as an intermediate cognitive process rather than a distal consequence.

To our knowledge, few studies have simultaneously examined psychological flexibility, fear of happiness, and life satisfaction within a single ACT-based model in young adults. By examining life satisfaction as a potential mediating mechanism, the present study extends prior research that has largely focused on bivariate associations and provides a more integrative, process-based understanding of how maladaptive responses to positive affect may emerge and be maintained.

Although prior research has often conceptualized fear of happiness as a predictor of life satisfaction and related well-being outcomes, this study adopts a complementary process-based perspective grounded in ACT. Rather than positioning global cognitive evaluations as primary determinants of emotional responses, ACT emphasizes the role of underlying regulatory processes, such as experiential avoidance, cognitive fusion, and reduced values-based action, in shaping how individuals relate to both negative and positive internal experiences. From this standpoint, reduced psychological flexibility is conceptualized as a proximal mechanism that influences not only how individuals evaluate their lives but also how they respond to positive affective states. Accordingly, life satisfaction is conceptualized here as an intermediate cognitive-evaluative domain that is more likely to reflect rather than determine individuals' responses to positive emotional experiences. This perspective extends existing models by examining whether process-based mechanisms precede and shape evaluative judgments, thereby contributing to a more nuanced and potentially bidirectional and dynamic understanding of the relationships among psychological flexibility, life satisfaction, and fear of happiness.

In line with this framework, the present study aims to examine the associations between psychological flexibility, fear of happiness, and life satisfaction in young adults, and whether life satisfaction functions as a mediating mechanism linking psychological flexibility and fear of happiness. Based on ACT theory and prior empirical findings, the following hypotheses were proposed:

**H1:** Psychological flexibility will be negatively associated with fear of happiness.
**H2:** Psychological flexibility will be positively associated with life satisfaction.
**H3:** Life satisfaction will be negatively associated with fear of happiness.
**H4:** Life satisfaction is proposed as a potential mediator of the relationship between psychological flexibility and fear of happiness.

From a theoretical perspective, clarifying the role of life satisfaction in this relationship contributes to a more nuanced understanding of how maladaptive responses to positive affect may emerge and be maintained. From a practical standpoint, identifying whether fear of happiness is more strongly associated with psychological flexibility or with broader life evaluations has important implications for intervention development. If maladaptive responses to positive emotions are more closely linked to reduced psychological flexibility, particularly experiential avoidance, interventions targeting psychological flexibility may be more effective than those focusing primarily on cognitive evaluations of life circumstances.

## 2 Method

### 2.1 Research design

This study employed a cross-sectional correlational design, in which variables were measured simultaneously at a single time point, allowing the examination of associations among variables while precluding causal inference (Çaparlar and Dönmez, 2016; Karasar, 2016). Accordingly, the present study focuses on examining associations and indirect effects among psychological flexibility, fear of happiness, and life satisfaction rather than establishing causal relationships.

The primary aim of the study was to examine the association between psychological flexibility and fear of happiness and to investigate whether life satisfaction functions as a mediating mechanism in this relationship. Given the cross-sectional nature of the data, the mediation model reflects indirect statistical associations rather than causal mechanisms (Hayes, 2022).

### 2.2 Participants

Participants were young adults aged 19–26 years who were currently enrolled in universities in Cyprus. Young adulthood represents a developmental period characterized by identity formation, increasing autonomy, and heightened sensitivity to social and evaluative experiences, thereby making this age group particularly relevant for examining psychological flexibility, fear of happiness, and life satisfaction (Arnett, 2000).

Participants were recruited using a convenience sampling strategy through online announcements distributed across multiple university departments to increase variability in academic background. Participation was voluntary, and no financial or academic incentives were provided.

Inclusion criteria were (a) being between 19 and 26 years of age, (b) being currently enrolled as a university student, (c) being single, and (d) not having children. These criteria were applied to ensure a relatively homogeneous developmental and relational context. Participants who did not meet these criteria were excluded from the study.

A total of 370 participants met the inclusion criteria and were included in the final analyses. The sample consisted of 235 women (63.5%) and 135 men (36.5%), with a mean age of 21.79 years (SD = 2.52). Participants were recruited from multiple universities in Cyprus, including the Northern Cyprus Campus of Ankara Social Sciences University, European University of Lefke, Eastern Mediterranean University, Near East University, Cyprus International University, Girne American University, University of Mediterranean Karpasia, and Cyprus Health and Social Sciences University.

An *a priori* power analysis was conducted using G^*^Power 3.1 (Faul et al., 2007) for a multiple regression model with five predictors, corresponding to the primary components of the moderated mediation model (predictor, mediator, moderator, and interaction terms). Assuming a medium effect size (*f*^2^ = 0.15), an alpha level of.05, and a desired power of.95, the analysis indicated that a minimum sample size of 138 participants would be required. The final sample size of 370 participants substantially exceeded this requirement, indicating that the study was adequately powered to detect the expected effects.

Ethical approval and informed consent procedures are described in the Procedure section. The demographic characteristics of the participants are presented in Table 1.

**Table 1:** Participant characteristics.

Variable	Category	*n*	%
Gender	Female	235	63.5
	Male	135	36.5
Socioeconomic status	High	145	39.2
	Medium	209	56.5
	Low	16	4.3

*N* = 370.

## 3 Measures

### 3.1 Personal information form

A personal information form was used to collect demographic information, including age, gender, educational background, academic department, and socioeconomic status. No identifying information was collected.

### 3.2 The acceptance and action questionnaire-II (AAQ-II)

The Acceptance and Action Questionnaire-II (AAQ-II; Bond et al., 2011) is a seven-item self-report instrument designed to assess psychological inflexibility. Items are rated on a seven-point Likert scale ranging from 1 (“never true”) to 7 (“always true”), with higher scores indicating greater psychological inflexibility. A sample item is “I am afraid of my feelings.” The Turkish adaptation of the AAQ-II has demonstrated satisfactory psychometric properties in terms of reliability and validity (Yavuz et al., 2016). In the present study, total scores were reverse-coded such that higher scores reflect greater psychological flexibility. Thus, psychological flexibility was used as the focal construct in the analyses. The internal consistency coefficient (Cronbach's alpha) for the current sample was.80.

### 3.3 Fear of happiness scale

The Fear of Happiness Scale (Joshanloo, 2013), adapted into Turkish by Demirci et al. (2016), consists of five items rated on a seven-point Likert scale ranging from 1 (“strongly disagree”) to 7 (“strongly agree”). The scale has a unidimensional structure, with higher scores reflecting greater fear of happiness. An example item is “I prefer not to be too joyful, because usually joy is followed by sadness.” In the present study, the internal consistency coefficient (Cronbach's alpha) was.90.

### 3.4 Satisfaction with life scale (SWLS)

The Satisfaction with Life Scale (SWLS; Diener et al., 1985), adapted into Turkish by Dagli and Baysal (2016), is a five-item self-report measure rated on a five-point Likert scale ranging from 1 (“strongly disagree”) to 5 (“strongly agree”). Higher scores indicate greater life satisfaction. An example item is “In most ways my life is close to my ideal.” In the present study, the internal consistency coefficient (Cronbach's alpha) was.82.

### 3.5 Procedure

Data were collected via an anonymous online survey. Prior to participation, all participants were informed about the aims of the study and provided informed consent electronically. Participation was voluntary, and participants had the right to withdraw from the study at any time without pesnalty.

Ethical approval for the study was obtained from the Scientific Research and Publication Ethics Committee (BAYEK) of Lefke Avrupa University (Approval Date: March 28, 2025; Decision No: BAYEK053.16). All procedures performed in this study involving human participants were conducted in accordance with the ethical standards of the institutional review board and with the 1975 Helsinki Declaration, as revised in 2013.

### 3.6 Statistical analysis

Data were analyzed using SPSS 27 based on responses from 370 participants. Prior to analysis, the dataset was screened for missing data, outliers, and violations of statistical assumptions. No missing data were observed, and no extreme univariate outliers were detected based on standardized *z*-scores (± 3.29). Descriptive statistics were computed for all study variables.

Normality was evaluated using skewness and kurtosis values alongside visual inspection of histograms and Q-Q plots. Skewness values ranged from −0.714 to 0.750 and kurtosis values ranged from −0.465 to 0.404, indicating no substantial deviation from normality and satisfying commonly accepted thresholds (± 2) (George and Mallery, 2010; Hair et al., 2019).

Multicollinearity diagnostics indicated high tolerance values (0.926) and low variance inflation factor (VIF) values (1.08), suggesting that multicollinearity was not a concern (Hair et al., 2019; Field, 2013).

Given that all data were collected from the same respondents at a single time point, common method bias was assessed using Harman's single-factor test. The first factor accounted for 34.2% of the total variance, indicating that common method bias was unlikely to substantially influence the findings; however, this result was interpreted cautiously due to the known limitations of this approach.

Bivariate associations were examined using Pearson correlation analysis. Moderated mediation analyses were conducted using PROCESS Model 59 with 5,000 bootstrap samples to estimate indirect effects and bias-corrected 95% confidence intervals (Hayes, 2022). Socioeconomic status was included as a covariate in all models, and continuous variables were mean-centered prior to the creation of interaction terms.

Given the cross-sectional design, the results of the mediation analyses were interpreted as reflecting indirect statistical associations rather than causal relationships.

## 4 Results

Descriptive statistics and bivariate correlations among the study variables are presented in Table 2. As shown in Table 2, psychological flexibility was positively associated with life satisfaction (*r* = 0.273, *p* < 0.001) and negatively associated with fear of happiness (*r* = −0.545, *p* < 0.001). Life satisfaction was also negatively correlated with fear of happiness (r = −0.198, *p* < 0.001). This relatively strong negative association provides initial support for the proposed role of psychological flexibility as a key factor in explaining variations in fear of happiness.

**Table 2:** Descriptive statistics and correlations among psychological flexibility, life satisfaction, and fear of happiness.

Variables	M	SD	1	2	3
1. Psychological flexibility	33.77	7.98	–		
2. Life satisfaction	2.99	0.77	0.273^**^	–	
3. Fear of happiness	2.87	1.59	−0.545^**^	−0.198^**^	–

*N* = 370. M = Mean; SD = Standard deviation. ^**^ *p* < 0.01.

In terms of effect sizes, the association between psychological flexibility and fear of happiness can be considered moderate to large, whereas the associations involving life satisfaction were small to moderate, indicating that life satisfaction may play a more limited direct role compared to psychological flexibility in explaining fear of happiness (Cohen, 1988). These findings provide preliminary support for the hypothesized relationships.

However, as bivariate correlations do not account for indirect or conditional processes, further analyses were conducted using a moderated mediation framework to examine whether this relationship operates through indirect and conditional processes.

### 4.1 Moderated mediation analysis

To examine whether the indirect effect of psychological flexibility on fear of happiness through life satisfaction varies as a function of gender, a moderated mediation analysis was conducted using PROCESS Model 59 (Hayes, 2022), controlling for socioeconomic status.

**Figure 1:**
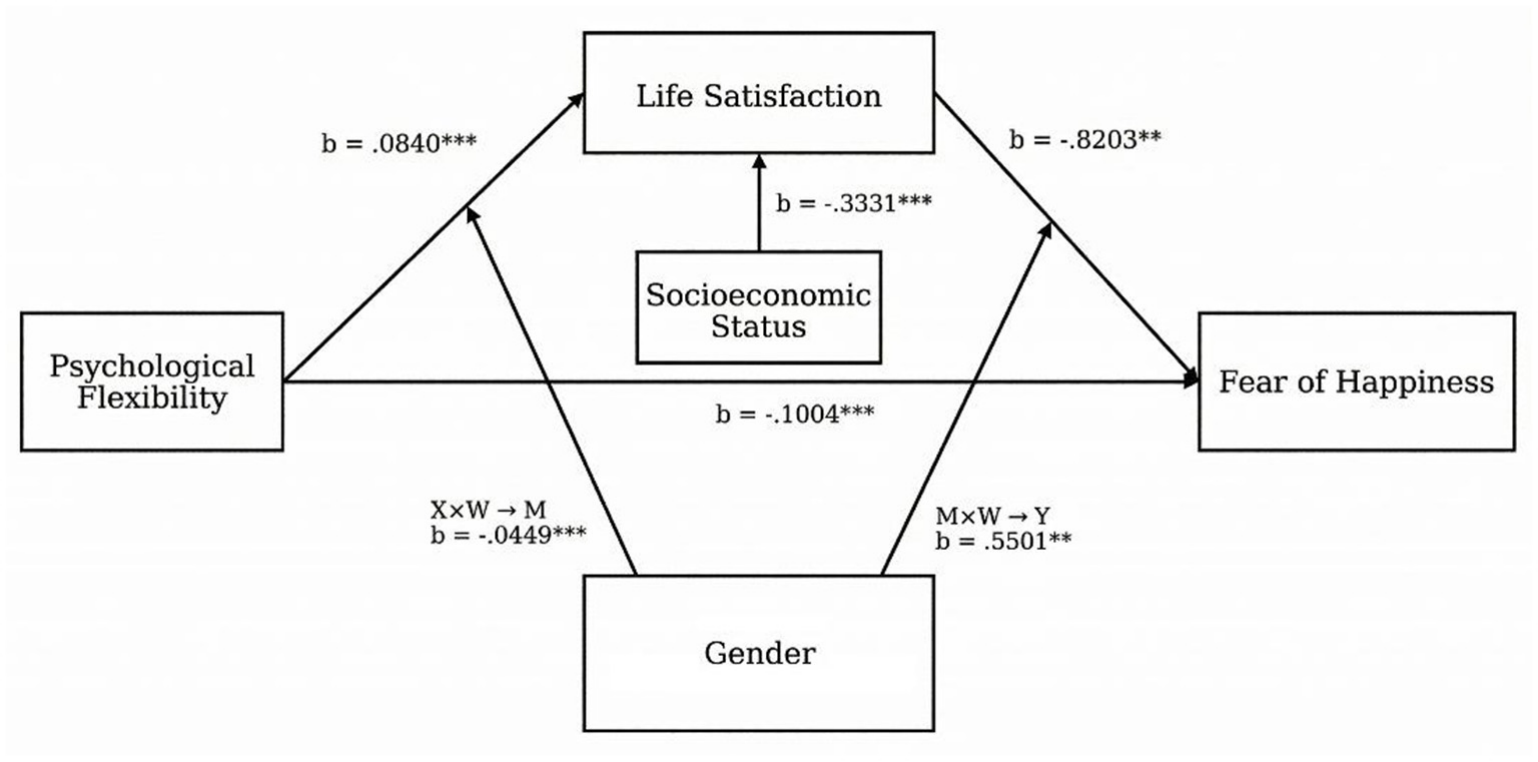
Moderated mediation analysis: the indirect effect of psychological flexibility on fear of happiness via life satisfaction as a function of gender. Unstandardized coefficients are reported. Socioeconomic status was included as a covariate in both the mediator and outcome models. All estimated paths are displayed. Gender (1 = women, 2 = men) was specified as a moderator of both the a path (psychological flexibility → life satisfaction) and the b path (life satisfaction → fear of happiness). *p* < 0.05, **p < 0.01, ***p < 0.001.

A conceptual representation of the tested model is presented in Figure 1. The results of the moderated mediation analysis are presented in the following sections.

### 4.2 Prediction of life satisfaction

The results of the regression model predicting life satisfaction are presented in Table 3. The overall model was statistically significant, *R*^2^ = 0.212, [F _(4, 365)_ = 24.61, *p* < 0.001], indicating that approximately 21.2% of the variance in life satisfaction was explained by the predictors. Psychological flexibility emerged as a significant positive predictor [*b* = 0.0840, SE = 0.0137, *t* = 6.13, *p* < 0.001, 95% CI (0.0570, 0.1109)].

**Table 3:** Prediction of life satisfaction.

Predictor	*B*	*SE*	*t*	*p*	*95% CI*
Psychological flexibility	0.0840	0.0137	6.13	< 0.001	[.0570, 0.1109]
Gender	1.197	0.3170	3.78	< 0.001	[.5735, 1.8204]
Psychological flexibility × gender	−0.0449	0.0091	−4.95	< 0.001	[−0.0628, −0.0271]
Socioeconomic status	−0.3331	0.0673	−4.95	< 0.001	[−0.4654, −0.2008]

Model: R^2^ = 0.212, F_(4, 365)_ = 24.61, *p* < 0.001; ΔR^2^ (interaction) = 0.053.

Gender (b = 1.197, *p* < 0.001) and socioeconomic status (b = −0.3331, *p* < .001) were also significant predictors. Importantly, the interaction between psychological flexibility and gender was statistically significant (b = −0.0449, SE = 0.0091, t = −4.95, *p* < 0.001), contributing an additional 5.3% to the explained variance (ΔR^2^ = 0.053), indicating a meaningful moderation effect.

Conditional effects analyses indicated that psychological flexibility significantly predicted life satisfaction among women (b = 0.0390, SE = 0.0060, *p* < 0.001), whereas this relationship was not significant among men (b = −0.0059, SE = 0.0071, p = 0.402). These findings indicate that gender moderates the association between psychological flexibility and life satisfaction. However, the presence of a significant effect in one group but not in the other does not, by itself, indicate a statistically significant moderation effect; therefore, the interaction term should be considered as the primary indicator of moderation.

### 4.3 Prediction of fear of happiness

The regression results for fear of happiness are presented in Table 4.

**Table 4:** Prediction of fear of happiness.

Predictor	*b*	*SE*	*t*	*p*	*95% CI*
Psychological flexibility	−0.1004	0.0287	−3.50	< 0.001	[−0.1568, −0.0441]
Life satisfaction	−0.8203	0.3040	−2.70	0.007	[−1.418, −0.2225]
Gender	−1.315	0.7629	−1.72	0.086	[−2.815, 0.1857]
Psychological flexibility × gender	0.000	0.0185	−0.00	0.999	[−0.0364, 0.0363]
Life satisfaction × gender	0.5501	0.1998	2.75	0.006	[0.1573, 0.9430]
Socioeconomic status	0.1020	0.1350	0.76	0.451	[−0.1635, 0.3675]

Model: R^2^ = 0.325, F _(6, 363)_ = 29.13, *p* < 0.001; ΔR^2^ (interaction) = 0.014.

The model was statistically significant, R^2^ = 0.325, F _(6, 363)_ = 29.13, *p* < 0.001, explaining 32.5% of the variance in fear of happiness. Psychological flexibility significantly and negatively predicted fear of happiness (b = −0.1004, SE = 0.0287, *t* = −3.50, *p* < .001), indicating that higher levels of psychological flexibility are associated with lower levels of fear of happiness.

Life satisfaction was also a significant negative predictor (b = −0.8203, SE = 0.3040, *t* = −2.70, *p* = 0.007). In contrast, gender (*p* = 0.086) and socioeconomic status (p =.451) were not significant predictors.

Regarding interaction effects, the interaction between psychological flexibility and gender was not significant (*p* = 0.999). However, the interaction between life satisfaction and gender was statistically significant (b = 0.5501, SE = 0.1998, *t* = 2.75, *p* = 0.006), contributing an additional 1.4% to the explained variance (ΔR^2^ = 0.014), indicating a modest moderation effect.

Conditional effects analyses indicated that life satisfaction significantly and negatively predicted fear of happiness among women (b = −0.2701, SE = 0.1329, *p* = 0.043), whereas this association did not reach statistical significance among men (b = 0.2800, SE = 0.1508, *p* = 0.064). However, the presence of a statistically significant effect in one group but not in the other does not, by itself, constitute evidence of moderation; therefore, the interaction term should be considered as the primary indicator of moderation.

Importantly, the direct effect of psychological flexibility on fear of happiness remained robust and statistically significant [b = −0.1004, SE = 0.0287, *t* = -−3.50, *p* < 0.001, 95% CI (−0.1568, −0.0441)], indicating that this relationship is largely independent of the mediating mechanism and represents the primary pathway through which psychological flexibility is associated with fear of happiness.

### 4.4 Conditional indirect effects

The conditional indirect effects of psychological flexibility on fear of happiness via life satisfaction are presented in Table 5.

**Table 5:** Conditional indirect effects.

Group	*Effect*	*BootSE*	*LLCI*	*ULCI*
Women	−0.0105	0.0060	−0.0233	−0.0001
Men	−0.0017	0.0038	−0.0106	0.0050

Bootstrapping analyses (5,000 resamples) indicated that the indirect effect was statistically significant for women [b = −0.0105, BootSE = 0.0060, 95% CI [−0.0233, −0.0001)], whereas the indirect effect was not statistically significant for men [b = −0.0017, BootSE = 0.0038, 95% CI (–.0106, 0.0050)].

However, the presence of a statistically significant indirect effect in one group but not in the other does not, in itself, constitute evidence of moderated mediation. Therefore, these findings should be interpreted cautiously and in conjunction with the index of moderated mediation, which provides a formal test of differences between conditional indirect effects across groups. In the present study, the index of moderated mediation was not statistically significant, indicating that the difference between groups was not statistically reliable.

### 4.5 Index of moderated mediation

The index of moderated mediation is reported in Table 6. The index of moderated mediation was not statistically significant [index = 0.0089, BootSE = 0.0071, 95% CI (−0.0049, 0.0238)]. This finding indicates that the difference between the conditional indirect effects across gender was not statistically reliable.

**Table 6:** Index of moderated mediation.

Index	*BootSE*	*LLCI*	*ULCI*
0.0089	0.0071	−0.0049	0.0238

*N* = 370. Unstandardized coefficients are reported. Percentile bootstrap confidence intervals were computed based on 5,000 resamples.

## 5 Discussion

The present study examined the relationship between psychological flexibility and fear of happiness, with particular attention to the mediating role of life satisfaction and the moderating role of gender. The findings indicate that psychological flexibility is a robust and consistent predictor of fear of happiness, whereas life satisfaction plays a relatively limited mediating role. Although gender influenced specific pathways within the model, the overall indirect effect did not significantly differ across groups.

Consistent with the preliminary analyses, psychological flexibility was negatively associated with fear of happiness, suggesting that individuals who are more open and adaptive in their engagement with internal experiences tend to report lower levels of apprehension toward positive emotions. Fear of happiness has been conceptualized as a belief system in which positive emotional experiences are perceived as potentially leading to negative consequences (Joshanloo, 2013; Joshanloo and Weijers, 2014). The present findings extend this perspective by demonstrating that such beliefs are more strongly associated with process-based regulatory mechanisms than with global evaluations of life.

### 5.1 The central role of psychological flexibility

The most prominent finding of this study is the significant direct effect of psychological flexibility on fear of happiness. This relationship remained statistically significant even after accounting for life satisfaction, gender, and interaction effects, suggesting that psychological flexibility represents a primary pathway through which individuals' responses to positive emotional experiences are shaped.

This finding is consistent with process-based models in contextual behavioral science, which conceptualize psychological flexibility as a core regulatory capacity that enables individuals to engage with internal experiences in an open and adaptive manner (Hayes et al., 2006; Kashdan and Rottenberg, 2010). From this perspective, fear of happiness appears to be closely associated with experiential avoidance processes, in which individuals respond to positive emotional experiences with hesitation or defensive processing (Chawla and Ostafin, 2007; Hayes et al., 1996).

Previous research suggests that avoidance of positive emotional experiences is linked to maladaptive regulatory processes, including discomfort with positive affect and difficulties in emotional integration (Gilbert et al., 2012; Kashdan et al., 2006). Importantly, individuals may develop avoidance tendencies not only toward negative internal experiences but also toward positive emotions. In this context, psychological flexibility may function as a protective factor by facilitating acceptance of positive experiences and reducing avoidance-based responses.

Taken together, these findings suggest that fear of happiness is more strongly associated with experiential and regulatory processes than with broader evaluative constructs. One potential mechanism underlying this relationship involves impaired regulation of positive affect, whereby individuals high in fear of happiness are more likely to dampen or suppress positive emotions rather than savor them (Joshanloo, 2024). Such patterns may limit engagement with positive experiences and reinforce avoidance-based regulatory tendencies.

Consistent with this perspective, prior research indicates that fear of happiness is associated with lower levels of resilience, which in turn predict reduced life satisfaction and flourishing (Yildirim, 2019). Extending this line of evidence, more recent findings demonstrate that fear of happiness is also linked to diminished psychological resources, including meaning in life and perceived social support, which collectively contribute to increased psychological distress (Yildirim et al., 2025). Within the framework of the broaden-and-build theory, these findings suggest that fear of happiness may inhibit the development of psychological resources by constraining individuals' engagement with positive emotional experiences.

From a process-based perspective, these results support the notion that fear of happiness operates within a broader regulatory system in which deficits in emotion regulation and experiential openness contribute to the emergence and maintenance of maladaptive responses to positive affect. Accordingly, psychological flexibility may function as an upstream regulatory mechanism that shapes individuals' responses to positive emotional experiences, thereby influencing downstream outcomes such as resilience and overall wellbeing.

Extending this process-based framework, the present findings indicate that psychological flexibility may influence fear of happiness through its role in shaping emotion regulation processes, particularly by reducing experiential avoidance and promoting openness to positive affect. In this sense, fear of happiness may reflect not only a cognitive belief system but also a broader behavioral and emotional tendency that restricts engagement with positive experiences and undermines adaptive psychological functioning.

The present findings are also consistent with experimental evidence demonstrating that psychological flexibility can be actively enhanced through ACT-based interventions, leading to improvements in emotional functioning and reductions in psychological distress (Köksal and Topkaya, 2025). This supports the interpretation that psychological flexibility operates as a dynamic and modifiable regulatory mechanism, influencing how individuals respond not only to negative experiences but also to positive emotional states such as happiness.

### 5.2 The limited mediating role of life satisfaction

Although life satisfaction was significantly associated with both psychological flexibility and fear of happiness, its mediating role was relatively limited. The indirect effect was small in magnitude and reached statistical significance only among women; however, this finding should be interpreted cautiously.

Importantly, the index of moderated mediation was not statistically significant, indicating that the indirect effect did not differ reliably across gender (Hayes, 2022). Therefore, the presence of a statistically significant indirect effect in one group but not in the other does not, in itself, constitute evidence of moderated mediation.

Conceptually, life satisfaction represents a global and cognitively mediated evaluation of one's overall life circumstances (Diener, 1984; Diener et al., 1985), whereas psychological flexibility reflects ongoing experiential and process-based regulatory capacities. This distinction is consistent with prior research suggesting that global cognitive evaluations may be less sensitive to dynamic and context-dependent emotional processes compared to process-oriented constructs such as experiential avoidance and emotion regulation (Ciarrochi et al., 2010; Ong et al., 2006).

Accordingly, the relatively limited mediating role of life satisfaction suggests that fear of happiness may be more strongly associated with how individuals relate to their internal experiences than with how they evaluate their overall life conditions. In addition, recent review evidence indicates that fear of happiness may influence how individuals respond to subjective well-being measures, including life satisfaction assessments, potentially functioning as a biasing factor in self-reported wellbeing (Joshanloo, 2024). This perspective may help explain why life satisfaction did not emerge as a strong mediating mechanism in the present study.

Furthermore, the present findings are consistent with broader evidence suggesting that fear of happiness operates through process-based mechanisms that may undermine adaptive psychological functioning. For example, previous research has shown that fear of happiness is associated with reduced resilience, lower meaning in life, and diminished perceived social support, indicating that it may disrupt the development and maintenance of key psychological resources (Yildirim et al., 2025). From this perspective, life satisfaction may represent a downstream outcome of these processes rather than a central mechanism through which psychological flexibility is associated with fear of happiness.

Taken together, these findings suggest that global evaluative constructs such as life satisfaction may play a more limited role in explaining the relationship between psychological flexibility and fear of happiness compared to more proximal, process-based variables. Accordingly, the present results highlight the importance of focusing on experiential and regulatory processes when seeking to understand maladaptive responses to positive emotional experiences.

Although alternative directional models—such as fear of happiness predicting life satisfaction—are theoretically plausible, the present study specifically tested a process-based pathway grounded in Acceptance and Commitment Therapy (ACT), emphasizing experiential regulation processes rather than outcome-based structures. Therefore, the findings should be interpreted within this theoretical framework.

Finally, given the cross-sectional design of the study, causal inferences cannot be drawn. Future longitudinal and experimental research is needed to clarify the temporal ordering of these variables and to examine whether the relationships observed in this study may reflect bidirectional or dynamic processes over time.

### 5.3 Contextual and covariate effects

The findings regarding gender provide a nuanced understanding of moderation effects. Gender significantly moderated both the relationship between psychological flexibility and life satisfaction (a path) and the relationship between life satisfaction and fear of happiness (b path). Specifically, psychological flexibility significantly predicted life satisfaction among women but not among men, and life satisfaction significantly predicted fear of happiness among women but not among men.

However, despite these pathway-specific effects, the index of moderated mediation was not statistically significant (Hayes, 2022), indicating that the overall indirect effect did not differ reliably across gender. This suggests that gender influences the strength of specific relationships within the model but does not substantially alter the underlying process linking psychological flexibility to fear of happiness.

These findings underscore the importance of distinguishing between path-specific moderation and model-level moderated mediation, as these represent conceptually and statistically distinct phenomena.

Socioeconomic status was included as a covariate and emerged as a significant negative predictor of life satisfaction, while it was not significantly associated with fear of happiness. This pattern suggests that socioeconomic conditions may influence individuals' overall evaluations of their lives but may be less directly related to their emotional responses to positive experiences.

This distinction is consistent with the conceptualization of life satisfaction as a construct shaped by broader contextual and structural factors (Diener, 1984), whereas fear of happiness appears to be more closely related to individual-level experiential and cognitive processes. Accordingly, socioeconomic status does not appear to play a central role in the primary relationship examined in this study.

### 5.4 Theoretical contributions and process-based implications

The present findings make several contributions to the literature. First, they underscore the central role of psychological flexibility in understanding fear of happiness, extending its relevance to the domain of positive emotional processes (Hayes et al., 2006; Kashdan and Rottenberg, 2010).

Second, the results support a process-based conceptualization of fear of happiness, suggesting that it may be more closely associated with experiential avoidance processes rather than being primarily driven by global cognitive evaluations (Chawla and Ostafin, 2007; Hayes et al., 1996).

Third, the findings refine the understanding of gender differences by demonstrating that moderation may occur at the level of specific pathways without necessarily resulting in differences in overall indirect effects (Hayes, 2022). This distinction may help clarify inconsistencies in prior research and aligns with emerging perspectives emphasizing the importance of differentiating between process-level dynamics and outcome-level evaluations in psychological models of wellbeing.

### 5.5 Limitations, methodological considerations, and future directions

Several limitations should be considered when interpreting these findings. A primary limitation relates to the measurement of psychological flexibility. In the present study, the Acceptance and Action Questionnaire-II (AAQ-II) was reverse-scored so that higher scores reflect greater psychological flexibility. However, the AAQ-II was originally developed to assess psychological inflexibility, with a primary focus on experiential avoidance, and may not adequately capture the multidimensional nature of psychological flexibility as conceptualized within the ACT framework. Psychological flexibility encompasses a broader set of processes, including acceptance, cognitive defusion, present-moment awareness, and values-based action. Accordingly, the findings should be interpreted with caution, as the operationalization used in this study may represent a proxy for flexibility rather than a comprehensive assessment of the construct.

In addition, the analyses were conducted using composite scores within an observed-variable framework; therefore, a full latent measurement model was not tested. Although the instruments used in this study have well-established psychometric properties, this approach does not account for measurement error. Accordingly, future research may benefit from employing latent variable modeling techniques to provide a more precise estimation of the relationships among constructs.

Second, the cross-sectional design limits the ability to draw causal inferences regarding the directionality of the relationships and constrains the examination of potential bidirectional associations among the variables. Although the proposed model is theoretically grounded, longitudinal and experimental designs are needed to clarify the temporal ordering of these constructs.

Third, all variables were assessed using self-report measures, which may introduce common method bias and response tendencies. Incorporating multi-method and multi-informant approaches in future studies would help strengthen measurement validity and reduce potential biases.

Fourth, the use of a general adult sample may limit generalizability to clinical or culturally diverse populations. Given that fear of happiness is influenced by cultural beliefs (Joshanloo, 2013), future research should examine these relationships across different cultural contexts.

Furthermore, prior research suggests that the association between fear of happiness and well-being may be bidirectional, with lower wellbeing potentially contributing to increased avoidance of positive emotional experiences (Joshanloo, 2024). This underscores the importance of employing longitudinal designs capable of capturing reciprocal and dynamic processes over time.

Finally, although a moderated mediation effect was not observed, this does not preclude the possibility that other mechanisms may be involved. Future research may explore alternative mediators, such as emotion regulation strategies, intolerance of uncertainty, or culturally shaped beliefs about positive emotions.

## 6 Conclusion

In conclusion, the present study demonstrates that psychological flexibility is a key factor associated with lower levels of fear of happiness, primarily through a direct relationship rather than through life satisfaction. Although gender moderates specific pathways within the model, it does not significantly alter the overall indirect effect.

These findings highlight the central role of experiential and process-based mechanisms in understanding fear of happiness and suggest that interventions aimed at enhancing psychological flexibility may facilitate more adaptive engagement with positive emotional experiences.

### 6.1 Conclusion and practical implications

The present findings highlight psychological flexibility as a central process underlying fear of happiness, demonstrating that individuals with higher levels of flexibility are less likely to respond to positive emotional experiences with fear-based or avoidant reactions. Notably, this relationship was primarily direct rather than mediated by life satisfaction, suggesting that fear of happiness is more closely linked to experiential and regulatory processes than to broader cognitive evaluations of one's life.

This pattern supports a process-based conceptualization of fear of happiness, indicating that maladaptive responses to positive emotions may stem from deficits in emotion regulation and experiential openness. Within this framework, psychological flexibility appears to function as an upstream regulatory mechanism that shapes individuals' engagement with positive emotional experiences and influences downstream outcomes related to wellbeing.

Although gender moderated specific pathways within the model, it did not significantly alter the overall indirect effect, underscoring the importance of distinguishing between pathway—specific moderation and model-level processes. In addition, the limited role of life satisfaction as a mediator suggests that fear of happiness may operate relatively independently of global life evaluations and instead reflect more immediate, experience-near regulatory processes.

Overall, these findings emphasize the importance of targeting experiential and regulatory mechanisms in interventions aimed at reducing fear of happiness. In particular, interventions grounded in Acceptance and Commitment Therapy may be especially effective in promoting adaptive engagement with positive emotional experiences by specifically reducing experiential avoidance and enhancing acceptance of positive affect.

## Data Availability

The raw data supporting the conclusions of this article will be made available by the authors, without undue reservation.
